# Study protocol: A multi-centre, double blind, randomised, placebo-controlled, parallel group, phase II trial (RIDD) to determine the efficacy of intra-nodular injection of anti-TNF to control disease progression in early Dupuytren’s disease, with an embedded dose response study.

**DOI:** 10.12688/wellcomeopenres.11466.2

**Published:** 2017-11-16

**Authors:** Jagdeep Nanchahal, Catherine Ball, Jennifer Swettenham, Susan Dutton, Vicki Barber, Joanna Black, Bethan Copsey, Melina Dritsaki, Peter Taylor, Alastair Gray, Marc Feldmann, Sarah Lamb

**Affiliations:** 1 Kennedy Institute of Rheumatology, University of Oxford, Roosevelt Drive, Oxford, OX3 7FY, UK; 2 Oxford Clinical Trials Research Unit, Centre for Statistics in Medicine, Nuffield Department of Orthopaedics, Rheumatology and Musculoskeletal Sciences, University of Oxford, Windmill Road, Oxford, OX3 7LDR, UK; 3 Nuffield Department of Orthopaedics, Rheumatology and Musculoskeletal Sciences, University of Oxford, Windmill Road, Oxford, OX3 7LDR, UK; 4 Health Economics Research Centre, Nuffield Department of Population Health, University of Oxford, Old Road, Oxford, OX3 7LF, UK

**Keywords:** Dupuytren’s disease, RCT, anti-TNF, fibrosis, adalimumab

## Abstract

Dupuytren’s disease is a common fibrotic condition of the hand affecting 4% of the population and causes the fingers to curl irreversibly into the palm. It has a strong familial tendency, there is no approved treatment for early stage disease, and patients with established digital contractures are most commonly treated by surgery. This is associated with prolonged recovery, and less invasive techniques have high recurrence rates. The myofibroblasts, the cells responsible for the excessive matrix deposition and contraction, are aggregated in nodules. Using excised diseased and control human tissue, we found that immune cells interspersed amongst the myofibroblasts secrete cytokines. Of these, only tumour necrosis factor (TNF) promoted the development of myofibroblasts. The clinically approved anti-TNF agents led to inhibition of the myofibroblast phenotype *in vitro*. This clinical trial is designed to assess the efficacy of the anti-TNF agent adalimumab on participants with early disease. The first part is a dose-ranging study where nodules of participants already scheduled for surgery will be injected with either placebo (saline) or varying doses of adalimumab. The excised tissue will then be analysed for markers of myofibroblast activity. The second part of the study will recruit participants with early stage disease. They will be randomised 1: 1 to receive either adalimumab or placebo at 3 month intervals over 1 year and will then be followed for a further 6 months. Outcome measures will include nodule hardness, size and disease progression. The trial will also determine the cost-effectiveness of adalimumb treatment for this group of participants.

## Introduction

### Background

Dupuytren’s disease (DD) is extremely common and is estimated to affect approximately 4% of the general UK and US populations ^1^. Between 35–50% of patients with early DD manifest as nodules on the palmar aspect of the hand go on to develop finger contractures ^2, 3^. The nodules are typically quiescent for a period and then become active, progressing to flexion deformities over a period of months ^4^. The mainstay of treatment remains surgical excision (fasciectomy) of the diseased tissue or cords ^5^, and recent US data show that ~60% of treated patients undergo surgery ^6^, with the remainder equally split between collagenase and needle fasciotomy. Generally, patients are offered these treatments once digits are flexed to 30 degrees or more and hand function is impaired ^7^. The recurrence rate in patients treated with surgery is 21% within 5 years ^8^, and these individuals may require more extensive surgery involving excision of the diseased tissue and overlying skin (dermofasciectomy). Post-operatively, patients require up to 6 months of hand therapy and splintage ^9^. Complications occur in approximately 20% of patients undergoing surgery for DD ^10, 11^. Alternative, less invasive techniques to disrupt the cords of diseased tissue with either a needle ^12^ or collagenase digestion ^13^ are associated with rapid recovery of hand function with minimal therapy ^14^. However, recurrence rates are high, at 5 years affecting 85% of patients treated with percutaneous needle aponeurotomy ^8^ and 47% of those treated with collagenase ^15^. The complication rate is 20% following needle aponeurotomy ^11^ and over 70%, where the majority are relatively minor and transient, mainly comprising swelling, bruising and pain after collagenase injection ^16^.

There is currently no approved therapy for the treatment of early DD. An uncontrolled and unblinded retrospective review of intralesional steroid injection at 6 week intervals in 63 patients with early DD reported that following a mean of 3.2 injections, there was subjective improvement of 60–80% in 97% of patients and disease reactivation occurred in 50% of patients 1 to 3 years after the last injection ^17^. Given the paucity and quality of data, this treatment modality has found limited acceptance. Similarly, studies reporting the efficacy of radiotherapy are limited by a lack of quality, with no blinding or randomisation and the use of subjective outcome measures. A recent systematic review of studies reporting outcomes in patients with early disease treated with radiotherapy ^18^ found that in four studies participant numbers were small (10 or fewer) ^19– 22^. Of the remaining 6 studies, 2 reported improvement ^23, 24^, 3 described equivocal results ^25– 27^ and one showed no change ^28^. One study noted that results following radiotherapy did not differ from the natural history of early DD ^27^. Approximately 20–30% of patients receiving radiotherapy in the studies included in the systematic review developed long term adverse effects, including dry skin, desquamation, skin atrophy, telangiectasia, erythema, and altered heat and pain sensation ^21– 23, 25– 27^. Based on the published data, NICE recommends that radiotherapy should only be used with special arrangements for clinical governance, consent and audit or in a research setting ^29^.

Therefore, there is a need to develop an effective therapy to retard progression of early DD and also prevent the development of recurrent disease following surgery, needle fasciotomy or collagenase injection in patients with established finger contractures.

Our laboratory studies based on tissues from patients with DD normally discarded at the time of surgery revealed the presence of innate immune cells, including macrophages, clustered in nodules ^30^. Freshly disaggregated cells from the nodules secreted a range of cytokines. The effects of these cytokines on contraction and profibrotic signaling pathways were assessed in fibroblasts from the palmar and non-palmar dermis of Dupuytren’s patients, and palmar fibroblasts from individuals without Dupuytren’s disease. Exogenous addition of TNF, but not other cytokines, including IL-6 and IL-1β, promoted differentiation only of palmar dermal fibroblasts from patients with Dupuytren’s disease into myofibroblasts via the Wnt signaling pathway. Neutralizing antibodies to TNF inhibited the contractile activity of myofibroblasts derived from Dupuytren’s patients, reduced their expression of α-smooth muscle actin, and mediated disassembly of the contractile apparatus. Of the anti-TNF agents approved for clinical use via subcutaneous administration, adalimumab and golimumab were found to be the most efficacious in downregulating the myofibroblast phenotype *in vitro* at the doses tested ^30^.

Based on these laboratory data, we are proceeding with a phase II clinical trial using adalimumab to assess the efficacy of intranodular injection for participants with early DD. Adalimumab is used to treat other conditions such as inflammatory arthritis and inflammatory bowel disease and has a well described safety profile. We conducted an end user survey of 46 patients, 24 with early DD and 22 who had previously undergone surgery. Thirty-three patients (71.7%) responded 62.5% (n=15) and 82% (n=18) respectively from each group. Our survey ( Table 1) indicated that both early disease and established disease patients would accept injection therapy that reduced need for future surgery.

**Table 1.** Summary of responses to questionnaire regarding acceptability of injection therapy that would retard the progression of disease. The survey was completed by 33 patients.

Extremely or very likely accept:	Patients with early Dupuytren’s disease (n=15)	Patients who had previously undergone surgery for Dupuytren’s disease (n=18)	Both cohorts combined (n=33)
1 injection per year for lifetime	14 (93%)	17 (94%)	31 (94%)
3 injections per year for lifetime	9 (60%)	12 (67%)	21 (64%)

### Objectives

The research hypothesis is to determine whether adalimumab injections control the progression of early Dupuytren’s disease more than placebo (saline). A two part trial has been designed to address these objectives.

## Study protocol

**Figure 1.**
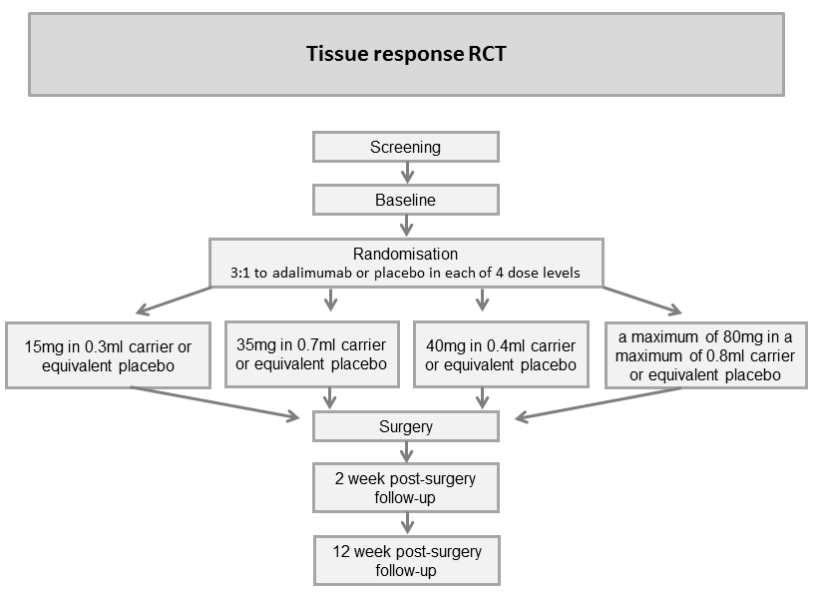
Flow chart: Tissue response RCT.

**Figure 2.**
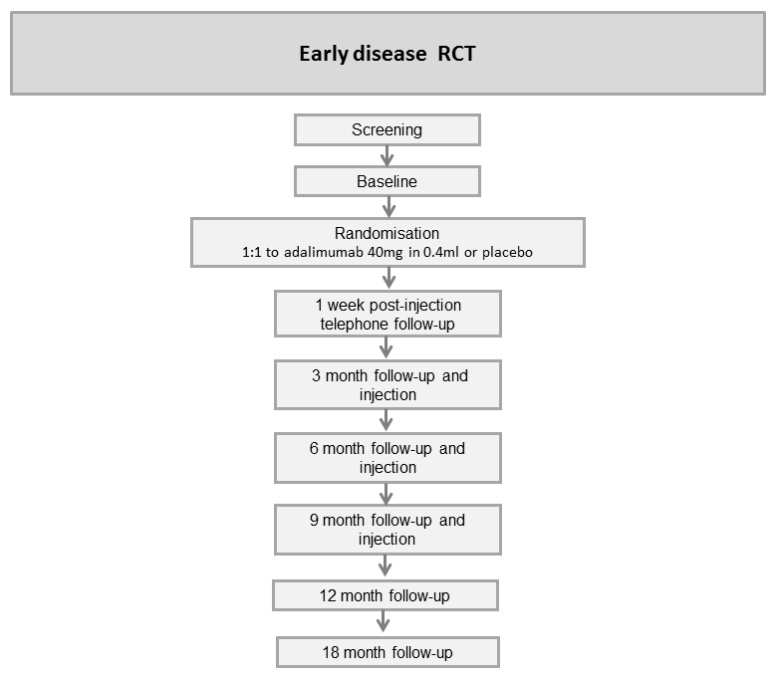
Flow chart: Early disease RCT.

The two part trial comprises:

1. Tissue response RCT: A single centre double blind randomised placebo-controlled trial (RCT) to determine the response of DD nodular tissue at the molecular level to escalating doses of adalimumab (anti-TNF) injected into the diseased tissue two weeks before planned surgical excision in participants with established DD ( Figure 1).
2. Early DD RCT: A multi-centre, double blind, randomised, placebo-controlled, parallel group, phase II RCT to determine the efficacy of intra-nodular injection of anti-TNF in controlling disease progression in participants with early DD ( Figure 2).

## 1. Tissue response RCT

**Study setting:** St John’s Hospital NHS Lothian

**Participants:** Participants with established DD leading to contractures ≥30° at either the metacarpophalangeal joint or the proximal interphalangeal joint and awaiting scheduled surgery of the hand for excision of the diseased Dupuytren’s tissue will be invited to join the study. Flexion deformities of ≥30° are associated with impairment of function and form the criteria used by the clinical commissioning groups for surgical referral. The diagnosis of DD is made on the basis of the history and clinical examination. Only a single nodule will be injected in each participant and the most prominent nodule due to be excised will be selected.

**Intervention:** Adalimumab delivered by intra-nodular injection 12–18 days prior to surgery in one of up to four different doses: 15mg in 0.3ml carrier, 35mg in 0.7ml carrier, 40mg in 0.4ml carrier (new carrier formulation) and a maximum of 80mg in a maximum of 0.8ml. A minimum of 8 participants will be recruited to each cohort, with up to a maximum of 40 participants in total. The last cohort will only be utilised if approved by the Trial Steering Committee (TSC) after a blinded interim analysis of the laboratory results.

**Control:** Injection of saline (placebo) of equivalent volume to intervention for each cohort.

**Outcomes:** Please see Table 2.

**Table 2.** Table of Tissue response RCT objectives and outcome measures.

	Objectives	Outcome measures
**Primary** **Objective**	1. To establish an effective dose of adalimumab for downregulating the myofibroblast phenotype	1. Expression of mRNA for α-SMA.
**Secondary** **Objectives**	2. To determine the effectiveness of adalimumab for myofibroblast inhibition.	2.1. Expression of mRNA for COL-1A1, COL-3A1 and cadherin 11. 2.2. Levels of α-SMA protein. 2.3. Hardness of selected nodule. 2.4. Ultrasound imaging of nodule size. 2.5. Adverse event assessment comparing active and placebo groups using visual inspection of injection site, surgery site and laboratory reports.
**Tertiary** **Objectives**	3. Determine circulating levels of adalimumab and antibodies to adalimumab in the blood. 4. To assess if DD injection therapy would be acceptable to participants 5. To evaluate the health-related quality of life of participants	3. Analysis of blood sample. 4. Analysis of injection experience questionnaire 5. Analysis of EQ-5D-5L data to estimate utilities using quality-adjusted life years (QALYs).

**Primary outcome:** Expression of mRNA for α-SMA to establish an effective dose of adalimumab for downregulation of the myofibroblast phenotype. 

**Secondary outcomes:** Expression of mRNA for COL-1A1, COL-3A1 and cadherin 11, and levels of α-SMA protein to determine the effectiveness of adalimumab for myofibroblast inhibition. Nodule hardness and ultrasound image of nodule dimensions/size. Adverse event assessment using visual inspection of injection site, surgery site and laboratory reports.

**Tertiary outcomes:** Circulating levels of adalimumab and antibodies to adalimumab. The injection experience will be rated by the participant and the injection site assessed for local adverse events.

**Data collection/follow-up summary:** Please see Table 3.

**Table 3.** Summary of schedule for Tissue response RCT.

	What will happen?	Is this visit combined with usual hospital treatment?
**Medical check-up** *Approx. 1–2 hours.*	Eligibility assessment including chest X-ray and blood tests.	**Yes**, combined with pre-operation assessment
**Injection visit** *Approx. 2 hours.*	• Dupuytren’s assessment • Blood test • Ultrasound scan • Hand photo • EQ-5D-5L Questionnaire • Nodule hardness • Injection and then assessment of injection site	**No**
**Surgery day** *Approx. 1 hour.*	• Assessment of injection site • Blood test • Ultrasound scan • Hand photo • Nodule hardness	**Yes**, while waiting for surgery
**2 weeks after** **surgery** *Approx. 20 mins.*	• Surgery site assessment. • Hand photo	**Yes**, combined with surgery aftercare
**12 weeks after** **surgery** *Approx. 15 mins.*	• EQ-5D-5L Questionnaire • Review of therapy record	**Yes**, combined with surgery aftercare

**Sample size:** Up to forty participants, with a minimum of 8 per cohort. Numbers are based on *in vitro* findings for the primary outcome measure, α-SMA expression of 0.55±0.11 on treatment with anti-TNF, compared to control gene expression of 1.03±0.18 in controls ^30^.

**Recruitment:** Participants with established DD and who are due to be scheduled for surgery for this disease will be identified and approached by hand specialists who will be assessing patients in an out-patient clinic. Potential participants will be given the relevant Participant Information Leaflet (PIL) containing a telephone number and an e-mail address to request further information.

**Randomisation:** 3:1 to adalimumab or placebo in each of the dose levels. Randomisation is computer-generated by the trial statistician. An allocation log will be stored securely in the pharmacy to indicate, for each trial ID, which investigational medicinal product (IMP) (drug or placebo) to dispense. Once consent is confirmed together with the dose cohort, the pharmacist dispenses the relevant treatment. The RRAMP system, an online system run by Oxford Clinical Trials Research Unit (OCTRU), will be used to store treatment allocations to enable emergency unblinding. 

**Blinding:** Participants and healthcare professionals involved in follow-up. Healthcare professional delivering injection also blind where possible (depending on formulation of adalimumab), as the 40mg in 0.4ml preparation of adalimumab is only available in a pre-filled syringe.

## 2. Early Dupuytren’s disease RCT

**Setting:** Multicentre; anticipated to run in three centres including Oxford NHS Hospitals Trust.

**Participants:** Participants with early DD nodules who also have shown or report progression of the disease in the previous 3–6 months will be invited to join the study. Only participants with flexion deformities of ≤30° at the metacarpophalangeal and/or at the proximal interphalangeal joint will be recruited, so total flexion deformity could be up to 60°. The participants will be randomised (1:1) to receive injections of either adalimumab or saline into the active nodule.

**Intervention:** Adalimumab 40mg in 0.4ml injected into the nodule at baseline, 3, 6 and 9 months after randomisation.

**Control:** Injection of saline (placebo) of equivalent volume.

**Outcomes:** Please see Table 4.

**Table 4.** Table of Early DD RCT objectives and outcome measures.

	Objectives	Outcome measures
**Primary** **Objective**	To determine if injection with adalimumab is superior to placebo injection of normal saline in controlling disease progression.	Hardness of selected nodule.
**Secondary** **Objectives**	1. To compare the development of Dupuytren’s nodules and associated cord, flexion deformities of the fingers and impairment of hand function for participants on each treatment. 2. Monitor for adverse events.	1.1. Ultrasound imaging of nodule size. 1.2. Range of motion of the affected digit. 1.3. Grip strength. 1.4. Participant Reported Outcomes: Michigan Hand Outcomes Questionnaire (MHQ) Participant identified activity most restricted by DD scored on a scale of 1–10. 1.5. Clinical assessment of the hand. 2.1. Adverse event assessment comparing active and placebo groups using visual inspection of injection site and laboratory reports. 2.2. Progression to surgery of the digit being assessed.
**Tertiary** **Objectives**	3. To assess if early DD injection therapy represents good value for money compared to current clinical care. 4. Monitor circulating levels of adalimumab and antibodies to adalimumab in the blood	3. Analysis of health care resource utilisation data and EQ-5D-5L data to estimate cost and utilities from participants on each treatment. 4. Analysis of blood sample.

**Primary outcome:** Nodule hardness measured using a tonometer.

**Secondary outcomes:** Nodule dimensions/area (determined by ultrasound imaging), range of motion of the affected digit, grip strength, patient reported outcome measures of hand function, progression to surgery of the digit being assessed, injection experience and adverse events.

**Tertiary outcomes:** Circulating levels of adalimumab, and antibodies to adalimumab. Healthcare resource use and health-related quality of life.

**Data collection/follow-up summary:** Please see Table 5

**Table 5.** Summary of schedule of events for early DD RCT.

	What will happen?
**Medical check-up** *Approx. 1-2 hours.*	Eligibility assessment including chest X-ray and blood tests.
**Injection visit 1** *Approx. 2 hours.*	• Dupuytren’s assessment • Blood test • Ultrasound scan • Hand photo • EQ-5D-5L and hand function questionnaires • Resource use questionnaire • Nodule hardness, finger movement and grip strength • Injection and then assessment of injection site
**Injection visit 2** **3 months after first injection** *Approx. 2 hours.*	• Assessment of nodules and cords in hand • Blood test • Ultrasound scan • Hand photo • EQ-5D-5L and hand function questionnaires • Resource use questionnaire • Nodule hardness, finger movement and grip strength • Injection and then assessment of injection site
**Injection visit 3 and 4** **6 and 9 months later** *Approx. 2 hours.*	• As at 3 months but without blood test
**12 months later** *Approx. 1 hour.*	• As at 3 months but without injection
**18 months later** *Approx. 1 hour.*	• As at 3 months but without injection or blood test

**Sample size:** For the early DD RCT, the sample size required is 138 participants based on detecting a standardised effect size of 0.62, at 5% significance (2-sided) and 90% power, allowing for a 20% loss to follow up. The target effect size was determined based on a 5 point change in nodule hardness at 12 months and assuming a standard deviation of 8. These estimates were based on data from a case-control pilot study ^31^. A pilot study of tonometry data from 25 patients with early DD demonstrated that the palmar tissues of patients with untreated early DD were significantly firmer (53±8) than the corresponding areas of 12 age and sex matched controls (32±3) when measured with a portable Rex Gauge durometer.

**Recruitment:** Participants will be recruited primarily from outpatient clinics, as well as advertisements in general practices local to trial sites and through relevant websites. Potential participants will be given/sent the PIL which contains a telephone number and an e-mail address to request further information or to make an appointment for consent and screening to participate in the study.

Potential participants will be contacted within 4 weeks of initial contact/expression of interest to arrange telephone screening or an invitation for further screening at clinic.

**Randomisation:** 1:1 adalimumab: placebo, computer generated stratified by age (18–49 or ≥50 years) and centre using RRAMP. RRAMP is used to randomise participants and this will generate a Trial ID and prompt an email to Pharmacy which informs them of which investigational medicinal product (IMP) (drug or placebo) to dispense. RRAMP also stores the treatment allocation enabling emergency unblinding if necessary.

**Blinding:** Participants and healthcare professionals involved in follow-up will be blinded. Those delivering the injection will not be blinded, as the 40mg in 0.4ml preparation of adalimumab is now only available in a pre-filled syringe.

## Participant eligibility criteria for both parts of the trial

All participants will give informed consent before eligibility is checked against the criteria listed in Table 6. The person obtaining consent will be suitably qualified and experienced, and authorised to do so by the Principal Investigator at each site. Participants must test negative for HIV, tuberculosis, and hepatitis B and C on serological testing and a chest X-ray, in accordance with local standard procedures for anti-TNF screening. Eligibility criteria are based on current clinical use of adalimumab for patients with rheumatoid arthritis ^32^ ( Table 6).

**Table 6.** RIDD trial eligibility criteria.

Inclusion criteria	
*	Tissue response RCT: DD affecting the fingers resulting in flexion deformities of ≥30° at the MCP and/or the PIP joint, with impaired hand function and awaiting surgery OR Early DD RCT: Participants with early DD nodules shown or reported to progress in the previous 6 months, with a flexion deformity of ≤30° at the metacarpophalangeal and/or at the proximal interphalangeal joint, i.e. total flexion deformity of up to 60° for the digit being assessed
*	Nodule distinct and identifiable
*	Adequate contraception during the study for women of child bearing potential or their male partners
*	Aged 18 years or above
*	Able and willing to comply with all study requirements
*	Willing to allow their general practitioner to be notified of study participation
*	Sufficient language fluency for informed consent and to complete questionnaires
**Exclusion criteria**	
*	Previous fasciectomy, dermofasciectomy, needle fasciotomy, collagenase injection, steroid injection to treat DD in the digit concerned, or previous radiotherapy to the hand concerned
*	Pregnant, lactating or planning pregnancy within 5 months after last injection
*	Significant renal or hepatic impairment
*	Scheduled procedures requiring general anaesthesia during the study, other than planned Dupuytren’s surgery (Tissue response RCT only)
*	Ever been diagnosed with cancer, is terminally ill or is inappropriate for placebo medication
*	Systemic inflammatory disorder such as rheumatoid arthritis or inflammatory bowel disease
*	Any other significant disease or disorder which, in the opinion of the Investigator, may either put the participants at risk because of participation in the study, or may influence the result of the study, or the participant’s ability to participate in the study
*	Participated in a study involving an investigational medicinal product in the past 12 weeks
*	Known allergy to any anti-TNF agent
*	Have HIV or hepatitis B or C. Participants in the Early DD RCT must not be at risk of hepatitis B infection
*	Known to have an infection or history of repeated infections
*	History of Tuberculosis (TB)
*	Multiple Sclerosis (MS) or other demyelinating diseases
*	History of local injection site reactions
*	Needle phobia
*	Moderate or severe heart failure
*	Being treated with coumarin anticoagulants, such as warfarin (Tissue response RCT only)
*	Known lung fibrosis
*	Being treated with concomitant biologic DMARDS
*	Have received a live vaccine within the previous 4 weeks. Participants may receive concurrent vaccinations but must avoid the use of live vaccines for 12 weeks after their last injection
*	Received parenteral steroids within the previous 6 weeks
*	Participants with known allergy to tetracaine or allergy to either lidocaine or prilocaine will not receive Ametop or EMLA cream anaesthetic, respectively

## Trial procedures for both parts of the trial

Participants will be recruited if they meet the inclusion criteria and provide written informed consent. All participants will receive a PIL. Written consent will be sought following a full verbal and written explanation of the trial. Participants will personally sign and date the current approved version of the Informed Consent Form before any trial specific procedures are performed. The person who obtained the consent will be suitably qualified and experienced, and authorised to do so by the Principal Investigator, at each site. A copy of the signed Informed Consent Form will be given to the participant.

## Screening

### Medical history and demographics

Relevant medical history and medications will be recorded, as well as demographic data including age, sex, smoking habits and alcohol consumption.

### Screening tests

To check for eligibility to anti-TNF therapy, blood tests and a chest X-ray will be performed. Participants will have a maximum of 20ml peripheral blood taken to screen for suitability for anti-TNF therapy according to local standard procedures. As a minimum this includes screening for Hepatitis B and C and HIV, and testing for latent TB. If a recent clear chest X-ray is not present in the patient’s records then a chest X-ray will be taken to screen for TB in accordance to local standard procedures for anti-TNF screening. If results are positive for any test, the participant will not be eligible to enter the trial and will be informed and counseled about the result of their tests.

### Baseline assessments

The maximum amount of time between screening and baseline is 8 weeks. For participants eligible after review of the results from the screening tests, the trial team will verbally check the participant is happy to continue to give consent for the trial and record any changes to their health since the screening visit.

Baseline assessments include:

• Ultrasound imaging to assess Dupuytren’s nodule size (greyscale).
• Clinical examination of the hand to establish baseline disease status, including participant reported disease duration, age at onset, occupation, family history of DD, the digits involved, configuration of the Dupuytren’s cords, joint involvement and whether the flexion deformities are fixed or can be passively corrected, presence of Garrod’s knuckle pads and Ledderhose’s disease of the feet.
• Tonometry measurement of the hardness of the palmar tissue and the underlying nodule.
• A digital photograph of the palm showing the selected nodule.
• Active and passive finger range of movement measured using a goniometer.
• A maximum of 12.5ml peripheral blood taken for assessment, in the central laboratory, of pre-treatment levels of adalimumab and antibodies to adalimumab.
• Participants will complete a Health Quality of Life Questionnaire, the EQ-5D-5L.
• The injection will be administered into a single Dupuytren’s nodule (see Injection section below). Participants will be offered application of Ametop gel or lidocaine/prilocaine cream/EMLA cream 30 minutes to 1 hour prior to injection.
• Injection experience questionnaire
• Injection site assessment by a trial healthcare professional blinded to treatment allocation to monitor for local adverse effects

In addition, for the early DD RCT only:

• The MHQ hand function questionnaire and identification of activity most restricted by DD, scoring it on a scale of 1–10.
• Grip strength measured using a JAMAR Dynamometer.

See Supplementary Table 1 for a summary of the schedule for Tissue response RCT and Supplementary Table 2 for the summary of schedule for early DD RCT

## Intervention

### Investigational medicinal product for both parts of the trial

Adalimumab is a human monoclonal antibody that has a Marketing Authorisation but will be used off-label for this study. Normal saline (0.9% NaCl) will be used as placebo. The safety profile of adalimumab is well known, with the most common adverse reactions being mild injection site reactions ^33^.

Where possible, the adalimumab will be supplied in a single use glass vial. However, a pre-filled syringe may be the only option if using the 40 mg in 0.4 ml formulation.

**Procedure for single use glass vials:** Participants, treating physicians and healthcare professionals involved with administering the IMP or administering any trial procedure from the injection onwards will be blinded to treatment.

The IMP (which will be stored in pharmacy) will be dispensed with accountability to a member of the research team who will take the IMP to a clinic room separate from the participant. In this separate room, a non-blinded member of the research team, who is not involved in administering the IMP or assessing the participant, will prepare and draw up the adalimumab or normal saline in a syringe according to the randomisation, and label the syringes with the participant’s ID. The label will not reveal the identity of the IMP. Both the IMP and placebo have a similar viscosity and appearance so that the two treatments, adalimumab or saline, will be indistinguishable. The syringe, without any identifying packaging, will be taken to the trial healthcare professional (blinded to treatment allocation) to inject the participant. Once adalimumab has been drawn up, it tends to lose potency and this precludes preparation of the syringes before the participant presents for treatment. There is no stipulated time limit. For this trial, no more than 1 hour will elapse before the injection is given.

**Procedure for pre-filled syringes:** Due to the distinctive appearance of the syringe, it is unlikely to be possible to blind the healthcare professional administering the injection to treatment allocation. Although this adds extra complication to the IMP administration, the benefits of using the new formulation make the efforts to maintain blinding worthwhile. The lower volume of the 40mgs in 0.4ml pre-filled syringe and removal of the excipient containing citrate should result in reduced pain and improved participant acceptability ^34, 35^. To protect the quality of the data, any person injecting with a pre-filled syringe will NOT be involved in administering any further trial procedures.

Any healthcare professionals involved with administering any trial procedure, including outcome assessments, after the injection of IMP will be blinded to treatment.

Participants will be blinded to treatment, using a physical screen.

The IMP (which will be stored in pharmacy) will be dispensed with accountability to a member of the research team who will take the IMP to a clinic room separate from the participant. A non-blinded member of the research team will prepare the saline injection for the placebo treatment allocation. All syringes (adalimumab or saline) will be labelled with the participant’s ID. Care will be taken to ensure any healthcare professionals blinded to treatment allocation are not present for the injection, and the syringe will be hidden from the participant’s view. A pre-filled syringe of adalimumab can be stored at up to 25°C for up to 14 days, therefore the one hour time limit described for glass vials is not applicable with pre-filled syringes.

**Tissue response RCT:** Consented participants will enter the trial and receive the injection two weeks prior to the scheduled surgery date. Intra-nodular injections will be delivered into the most prominent nodule due to be excised by surgery. If participants do not proceed to surgery at 12–18 days following injection, the reason will be recorded and they will continue with normal care. These participants may be replaced if still during the recruitment phase of the tissue response RCT. Participants may withdraw from the study and continue normal NHS care. If possible, data collected up to the point of withdrawal will be retained, and if the participant has received the injection then a safety review of the participant medical notes will be undertaken at 3 months from randomisation.

**Early DD RCT:** Consented participants will be screened and if eligible, after consent is reaffirmed, they will be randomised (1:1) to receive injections of adalimumab (40mg in 0.4ml carrier) or saline (placebo 0.4ml) into the active nodule. The participants will receive further injections on a maximum of 4 occasions: baseline, 3, 6 and 9 months with further follow-up at 12 and 18 months with no injections. Injections will not be administered if the treated nodule decreases in size such that there is insufficient nodule tissue to inject. In this case, the participant will be encouraged to continue for all intervention visits and injections may be reintroduced if the nodule recurs. Injections will be discontinued if the participant withdraws from the trial or if the investigator considers it necessary for any reason, including pregnancy, adverse events, significant protocol deviation or non-compliance with study procedures. Compliance will be defined as attending not less than 75% of injection visits, i.e. 3 visits.

Participants may withdraw from the study and continue normal NHS care. If possible, data collected up to the point of withdrawal will be retained, and if the participant has received an injection then a safety review of the participant medical notes will be undertaken for up to 18 months from randomisation.

## Trial outcome measures

**Tissue analysis (Tissue response RCT only):** The injected nodule and surrounding diseased tissue will be removed during scheduled surgery approximately two weeks after treatment. The excised nodule tissue will be transported as per instructions specified in the trial Sample Handling Manual to the Kennedy Institute laboratories in Oxford, where tissue analyses will be undertaken. A scientist not involved with the clinical assessment and blinded to treatment allocation will dissect the nodule and extract the mRNA and protein. rt-PCR (reverse transcription polymerase chain reaction) will be carried out as previously described ^30^. Protein levels will be measured using electrochemiluminescence (Meso Scale Diagnostics, Maryland, USA) or Western blotting (Verjee et al., 2013).

Tissue samples and derivatives will be stored in the Kennedy Institute laboratory at the Botnar Research Centre in secure -80°C storage. The laboratory has undergone a self-assessment for good clinical practice in line with the UKCRC ‘Self-Assessment Questionnaire for assessing regulatory compliance in laboratories that perform the storage and analysis or evaluation of research samples’ ^36^. 

## Clinical outcome measures

**Nodule hardness (tonometry):** Will be measured by a portable Rex Gauge durometer RX-1800-00 (Rex Gauge Company Inc. Illinois, U.S.A.). Tissue hardness will be measured with a tonometer, ensuring that the gauge is balanced perpendicular to the skin without application of external pressure by the observer. Five readings will be obtained on each occasion. A photograph obtained at base line marking the spot to be measured will be used for all subsequent measurement to ensure consistency of placement of the tonometer. Tonometry has been used to evaluate skin compliance before and after surgery ^37^ and reported softening of the palm following excision of Dupuytren’s tissue.

**Grip strength (Early DD RCT only):** Grip strength will be measured using a Jamar meter (CE marked)

**Range of motion of the affected digit:** Individual range of movement of each joint in the affected digit will be measured using a goniometer.

**Clinical assessment of the hand:** Will assess any changes in the injected nodule and adjacent cord.

**Nodule size and vascularity:** Nodule size and vascularity will be assessed by an ultrasound scan with the ultrasound probe placed centrally over the nodule. The ultrasound will produce a quantitative result using ImageJ.

## Participant reported outcome measures

**Injection experience questionnaire:** Participants will report on the experience of each research injection using an injection questionnaire which incorporates a numeric rating scale.

**Participant identified activity most restricted by DD scored on a scale of 1–10 (Early DD RCT only):** Participants will be asked to identify at baseline the activity most restricted by their DD on a scale of 1–10. This activity will be reassessed at all follow-up timepoints.

**Michigan Hand Outcomes Questionnaire (MHQ) (early DD RCT only):** The MHQ ^38^ is a hand-specific validated outcome measure that, unlike some other instruments, allows the user to separately score each hand. It takes approximately 15 minutes to complete and comprises six distinct scales that assess overall hand function, activities of daily living, pain, work performance, aesthetics, and satisfaction with hand function. The MHQ score ranges from 0–100, with higher scores indicating better hand performance and a higher pain score indicating more pain. The MHQ is a validated outcomes measure that has been shown to be sensitive to change when used to assess improvement in hand function following surgery for established DD ^39, 40^ and was selected following a systematic review of outcome measures for DD ^41^.

**Health utilities using EQ-5D-5L:** The EQ-5D-5L is a validated, generalised, health related quality of life questionnaire recommended by the National Institute for Health and Care Excellence (NICE) as the accepted measure for conducting a cost-utility analysis. The EQ-5D-5L has been used to calculate health state utilities ^42^ in individuals with more advanced DD. The EQ-5D instrument facilitates the generation of a utility score from a person’s health related quality of life ^43^. A utility score refers to the preference that individuals have for any particular set of health outcomes. The EQ-5D consists of five health state dimensions (mobility, self-care, usual activity, pain/discomfort, and anxiety/depression). There are three levels of health status: no problems, some problems, major problems. Each participant will value their present health at the date of questionnaire completion. Participants will also complete a Visual Analogue Scale which will provide us with a value for the participant’s self-rated health at the time of survey completion.

Permission has been granted for the use of the MHQ and EQ-5D-5L from the relevant agencies.

**Resource use questionnaire (early DD RCT only):** Participant self-reported information on service use will be collected at 3, 6, 9, 12 and 18 months post randomisation to capture the intensity of use of different healthcare services, including primary, community and social care. Unit cost data will be obtained from national databases such as the BNF and PSSRU Costs of Health and Social Care ^44^. Where these are not available the unit cost will be estimated in consultation with the lead hospital finance department. In the latter process a blanket cost will be applied to the items used to carry out the intervention, using a base case provided by the Oxford University Hospital NHS Trust. No additional consent is required for the acquisition of these data. Participants will also have the opportunity to detail their out of pocket expenditure related to their treatment, as well as their ability to return to paid work after treatment.

## Safety monitoring

All adverse events (AEs) graded 3 and above according to the Common Terminology Criteria for Adverse Events (CTCAE) v4.0 ^45^ occurring during the trial and up to 12 weeks after surgery (Tissue response RCT) or until the end of participation (Early DD RCT) that are observed by the Investigator or reported by the participant will be recorded on the case report form (CRF), whether or not they may be attributed to trial medication. AEs considered related to the trial medication as judged by a medically qualified investigator will be followed either until resolution, or until the event is considered stable. Serious adverse events (SAEs) will be recorded on the trial specific SAE form and reported within 24 hours of the Site Study Team becoming aware of the event. Causality will be assessed by a medically qualified doctor. The Trial Co-ordinating Centre will be responsible for assessing causality and expectedness. Any SAEs deemed to be suspected unexpected serious adverse reactions (SUSARs) will be reported to the sponsor, the Medicines and Healthcare products Regulatory Agency (MHRA) and ethics committee within required timelines.

**Injection site assessment:** The most common adverse reaction with adalimumab is injection site reactions (erythema and/or itching, haemorrhage, pain or swelling). Adverse event assessment will involve visual inspection of injection site, surgery site and laboratory reports. The injection site will be monitored for adverse events using the trial injection site response form. The surgery site and subsequent scar will be monitored using a validated measure for wound and scar assessment: POSAS ^46^.

**Progression to surgery of the digit being assessed (Early DD RCT only):** Progression to surgery of the digit being assessed, if applicable, will be recorded during the 18 month follow-up. Progression to treatment will be determined by the clinicians, who are not part of the trial team.

**Monitor blood circulating levels of adalimumab and antibodies to adalimumab:** 12.5ml of blood will be collected pre- and post-injection to measure circulating levels of adalimumab and antibodies to adalimumab. Monitoring circulating levels of adalimumab will facilitate our understanding of the kinetics of drug absorption following intranodular injections and may provide further information regarding the optimal frequency of intranodular injection.

## Data management

A detailed Data Management Plan (DMP) will be followed for the management and monitoring of data. All trial data will be entered on paper CRFs, sent to the RIDD trial office and entered centrally into the trial database. The participants will be identified by a unique trial specific number in a database. Participant identifiable information will be securely stored in a locked cabinet onsite with restricted access, separately from the clinical trial database.

### Biological samples

Tissue samples and derivatives will be stored in the Kennedy Institute laboratory at the Botnar Research Centre in secure -80°C storage. Raw data will be analysed and all data, including metadata, will be stored on the University of Oxford servers. Summary data will be transferred to the trial database. Full details will be recorded in the DMP.

## Statistics

A separate statistical analysis plan (SAP) with full details of all statistical analyses planned for the data of this study will be drafted early in the trial and finalised prior to any primary outcome analysis. The SAP will be reviewed and will receive input from the Trial Steering Committee (TSC) and the Data and Safety Monitoring Committee (DSMC). The analysis will be undertaken using R, STATA (StataCorp LP), or other well-validated statistical packages.

Descriptive statistics will be used to describe the demographics between the intervention groups. Continuous outcome measures will be presented as a difference in means, together with a 95% confidence interval (CI) for each dose cohort (where applicable) and overall. Binary and categorical outcomes will be presented as numbers and percentages in each category, as well as the difference in proportions with the corresponding 95% CI.

The primary outcome for the Tissue Response RCT is expression of mRNA for α-SMA, quantified with PCR using the standard curve method using three housekeeping genes GAPDH, B2M, and PGK1 to normalize the samples. For analysis of the primary and key secondary outcomes the placebo results will be pooled across cohorts to allow for a dose response to be explored. For the safety outcomes, they will be reported within their respective cohorts due to the different volumes being used.

For the primary outcome of the early DD RCT, the difference in the mean change of nodule hardness between the two groups will be reported with 95% confidence intervals. The comparison of change in nodule hardness between interventions will be analysed using Analysis of Covariance (ANCOVA), adjusting for baseline if it is normally distributed. Otherwise, an equivalent non-parametric unadjusted test will be used. In order to analyse change in nodule hardness over the full-time period, ANCOVA will be used to compare nodule hardness between interventions utilising all time-points up to 18 months and adjusting for stratification and other important prognostic factors. For all other continuous variables, t-tests (or ANCOVA) will be applied if normally distributed to compare the intervention with the control group and the difference in the means, and the corresponding 95% confidence interval will be reported. If not normally distributed, non-parametric techniques will be used. For categorical variables, chi-squared tests will be used for comparing intervention groups if the variables are normally distributed. If a variable is not normally distributed, a non-parametric test will be used for the analysis. The level of significance to be used is p≤0.05.

The primary statistical analysis will be carried out on the basis of intention-to-treat, with all randomised participants included and analysed according to their allocated treatment group, irrespective of which treatment they actually received. Due to the small sample size, missing data will not be imputed for the tissue response RCT. For the primary outcome of the early DD RCT, multiple imputation using multivariate normal imputation, or other appropriate techniques, will be used to impute missing data if required (full details will be specified in the SAP). For the early DD RCT, sensitivity analyses will be run on the per protocol population which will be defined in the SAP.

Consistency of results will be compared across stratification factors using interactions and displayed in Forest plots; however, no formal tests will be performed by subgroup.

A decision analytical Markov model combining information on costs, quality of life and transition probabilities associated to each health state for the first and second year post-treatment will be used to estimate the long-term cost-effectiveness of anti-TNF therapy compared to current clinical practice in early DD for the Early DD RCT.

## Trial committees

### 1. Trial Management Group (TMG)

The TMG will be responsible for day-to-day management of the research for the Tissue response RCT and the Early DD RCT. Members will include members of the research team, the chief investigator, trial manager and statistician.

### 2. Safety Committee (SC) Tissue response RCT

The Safety Committee will include members of the TMG and an independent clinician.

The aims of this committee are to review incoming safety data and to make recommendations to the PI regarding dose selection, or to the TSC if the safety data indicate the trial should be terminated.

### 3. Data and Safety Monitoring Committee (DSMC)

The DSMC will review recruitment, study conduct and participant safety in the Early DD RCT and will consist of at least two independent clinicians and a statistician. This committee will review the accumulating data some of which will be analysed separately by treatment arm. They will make recommendations to the TSC. The terms of reference will be according to a DSMC charter based on the DAMOCLES recommendations ^47^.

### 4. The Trial Steering Committee (TSC)

The TSC will be an independent committee who are ultimately responsible for making decisions about the continuation or otherwise of the trial. The TSC will oversee the whole trial and will have the overall responsibility on decisions to continue or stop early. The terms of reference will be according to a TSC charter.

## Trial organization and administration

The study has received approval from the South Central - Oxford B Research Ethics Committee and the MHRA, and will be carried out following local legal and regulatory requirements. The trial is registered with the European Clinical Trials Database (EudraCT: 2015-001780-40) and has the Ethics Reference: 15/SC/0259. All substantial amendments to the protocol will be submitted to the ethics committee for approval. The study is sponsored by the University of Oxford.

All procedures relating to the trial and personnel involved will be carried out in accordance with Medical Research Council Clinical Practice, applicable UK legislation and the ethical principles of the Declaration of Helsinki. This study protocol follows SPIRIT guidelines ^48^. The trial will be reported in line with the CONSORT statement ^49^.

## Discussion

Dupuytren’s disease is a very common age dependent fibrotic disorder of the hand and the number of patients requiring treatment in the UK is expected to increase by 50% by 2030 ^50^. The mainstay of treatment remains surgical excision but requires prolonged post-operative hand therapy, may be associated with a relatively high complication rate and is not cost effective ^51, 52^. Less invasive procedures such as disruption of the cord with a needle or collagenase are associated with rapid recovery of function but have much higher recurrence rates ^53, 54^. The ideal treatment would prevent the progression of early nodular disease, before the development of digital flexion contractures which lead to impairment of hand function. Whilst a number of other treatments, including local steroid injection and radiotherapy, have been reported, evidence for efficacy is lacking and they have not been approved or gained widespread acceptance ^18^.

Our laboratory data showed that DD is a localised inflammatory disorder ^30^. The nodular tissue comprises mainly myofibroblasts, which both secrete and contract the matrix, leading to digital contractures ^55^. Pro-inflammatory cytokines are secreted by the infiltrating immune cells and we have found that only TNF leads to the differentiation of precursor cells into myofibroblasts, and that TNF inhibition downregulates the myofibroblast phenotype ^30^. These findings form the basis of our current clinical trial, where we will recruit participants with early stage DD to receive either adalimumab or saline at 3 monthly intervals injected directly into the nodules over a 9 month period and followed up for a further 9 months. The majority of patients with flexion deformities of the digits are treated by surgery. This provides the opportunity to obtain tissue for analysis following injection of the IMP. Therefore, we will recruit individuals scheduled for surgical excision of DD, administer adalimumab or placebo two weeks before surgery and determine the effect on the myofibroblast phenotype in the surgically excised tissues. This part of the trial will assess the efficacy of different doses of adalimumab at the mRNA and protein level to inform the molecular mechanism of action of anti-TNF in DD. The myofibroblast is responsible for all forms of fibrosis ^56^ and the data from this trial may inform future studies on other fibrotic diseases such as frozen shoulder.

**Study publication policy:** The Investigators will be involved in reviewing drafts of the manuscripts, abstracts, press releases and any other publications arising from the study. Authorship will be determined in accordance with the International Committee of Medical Journal Editors (ICMJE) guidelines and other contributors will be acknowledged.

## Supplementary material

Supplementary Table 1. Summary of schedule for Tissue response RCT.

Supplementary Table 2. Summary of schedule for early DD RCT.
