# Supplementary material for: Study protocol: A multi-centre, double blind, randomised, placebo-controlled, parallel group, phase II trial (RIDD) to determine the efficacy of intra-nodular injection of anti-TNF to control disease progression in early Dupuytren’s disease, with an embedded dose response study
Source: Wellcome Open Res. 2017 Nov 16;2:37. Originally published 2017 Jun 6. [Version 2] doi: 10.12688/wellcomeopenres.11466.2 (PMC5701439; doi:10.12688/wellcomeopenres.11466.2)
Supplement: Supplementary file 1 [file wellcomeopenres-2-14272-s0000.tgz › bb45a6b6-0213-49b2-b2dc-180b967f5d49.pdf]

Supplementary Table 1. Summary of schedule for Tissue response RCT.

**Procedure**	Time in mins	Screening Visit 1	Baseline Visit 2 Week 0	Phone call Week 1	Visit 3 Week 2	HAND SURGERY	Visit 4 Week 2 post-surgery	Visit 5 Week 12 post-surgery
Visit window					±3 days		±1 week	±4 weeks
Informed consent	20	**X**						
Demographics	5	**X**						
Medical history	5	**X**						
Concomitant medications	5	**X**	**X**		**X**		**X**	**X**
Physical examination	5		**X**					
Chest X-Ray	20	**X**						
Blood for screening	5	**X**						
Blood for research	5		**X**		**X**			
Dupuytren's assessment, inc. range of motion	15		**X**					
Eligibility assessment	5	**X**	**X**					
Health questionnaire	5		**X**					**X**
Nodule hardness (tonometry)	5		**X**		**X**			
Ultrasound imaging	15		**X**		**X**			
Digital photograph of palm	2		**X**		**X**		**X**	
Randomisation			**X**					
Topical anaesthetic and Injection of study drugs or placebo	40		**X**					
Injection questionnaire	5		**X**					
Adverse event assessment Injection site assessment	5		**X**		**X**			
Adverse event assessment Surgery site assessment	5			**X**			**X**	**X**
**Total in minutes**		**65**	**112**	**5**	**37**		**12**	**15**
