# Supplementary material for: Study protocol: A multi-centre, double blind, randomised, placebo-controlled, parallel group, phase II trial (RIDD) to determine the efficacy of intra-nodular injection of anti-TNF to control disease progression in early Dupuytren’s disease, with an embedded dose response study
Source: Wellcome Open Res. 2017 Nov 16;2:37. Originally published 2017 Jun 6. [Version 2] doi: 10.12688/wellcomeopenres.11466.2 (PMC5701439; doi:10.12688/wellcomeopenres.11466.2)
Supplement: Supplementary file 2 [file wellcomeopenres-2-14272-s0001.tgz › fe5a7347-baba-427a-ada2-47da61680f86.pdf]

Supplementary Table 2. Summary of schedule for early DD RCT.

**Procedures**		**Visits**							
	Time in minutes	Screening Visit 1	Baseline Visit 2 week 0	Phone call week1	Visit 3 3 month	Visit 4 6 month	Visit 5 9 month	Visit 6 12 month	Visit 7 18 month
Visit window				±4 days	±2 weeks	±4 weeks	±4 weeks	±4 weeks	±4 weeks
Informed consent	20	**X**							
Demographics	5	**X**							
Medical history	5	**X**							
Concomitant medications	5	**X**	**X**		**X**	**X**	**X**	**X**	**X**
Physical examination	10		**X**						
Chest X-Ray	20	**X**							
Blood for screening	5	**X**							
Blood for research	5		**X**		**X**			**X**	
Dupuytren's assessment	10		**X**						
Eligibility assessment	5	**X**	**X**						
Health questionnaire	5		**X**		**X**	**X**	**X**	**X**	**X**
Hand function questionnaires	10		**X**		**X**	**X**	**X**	**X**	**X**
Physical measures: grip, range of motion, tonometry	15		**X**		**X**	**X**	**X**	**X**	**X**
Ultrasound imaging	15		**X**		**X**	**X**	**X**	**X**	**X**
Digital photograph of palm	2		**X**		**X**	**X**	**X**	**X**	**X**
Randomisation			**X**						
Topical anaesthetic and Injection of study drugs or placebo	40		**X**		**X**	**X**	**X**		
Injection questionnaire	5		**X**		**X**	**X**	**X**		
Adverse event assessment Injection site assessment	5		**X**	**X**	**X**	**X**	**X**	**X**	
**Total in minutes**		**75**	**132**	**5**	**107**	**102**	**102**	**57**	**52**
