# Supplementary material for: Study protocol: A multi-centre, double blind, randomised, placebo-controlled, parallel group, phase II trial (RIDD) to determine the efficacy of intra-nodular injection of anti-TNF to control disease progression in early Dupuytren’s disease, with an embedded dose response study
Source: Wellcome Open Res. 2017 Nov 16;2:37. Originally published 2017 Jun 6. [Version 2] doi: 10.12688/wellcomeopenres.11466.2 (PMC5701439; doi:10.12688/wellcomeopenres.11466.2)
Supplement: Peer review history

# Peer review: Referee response for version 2

**DOI:** 10.21956/wellcomeopenres.14272.r28067

**Published:** 2017-11-23

**Authors:** David Warwick

**Affiliations:** 1 Orthopaedics, University Hospital Southampton, Southampton, UK; 2 Faculty of Medicine, University of Southampton, Southampton, UK

May I thank the Authors for the considerable effort they have made in responding to my suggestions and concerns. The paper is now, in my opinion, suitable to progress to indexing.

I have read this submission. I believe that I have an appropriate level of expertise to confirm that it is of an acceptable scientific standard.

# Peer review: Referee response for version 1

**DOI:** 10.21956/wellcomeopenres.12379.r23290

**Published:** 2017-06-27

**Authors:** David Warwick

**Affiliations:** 1 Orthopaedics, University Hospital Southampton, Southampton, UK; 2 Faculty of Medicine, University of Southampton, Southampton, UK

**STRONG POINTS**

- The Authors present a fascinating and plausible attempt to modify this enigmatic disease.
- The study design is well-developed and appropriate for the matter in question.

 

**POINTS TO IMPROVE**

- There does seem to be some misunderstanding of the difference between a nodule and a cord. Yet this distinction is essential. Surely the whole purpose of the study is to examine whether one can prevent a nodule from becoming a cord? This point needs to be fully addressed.
- In the Introduction and Discussion, there seems to me to have been just a little adornment in the quest to depict the need for this study: the portrayed risks of progression and complications of existing treatment, the alleged lack of cost effectiveness of treatment and the suggested need for prolonged hand therapy.
- The facts in the introduction should therefore be qualified
   
  - RCT evidence shows that splintage is not needed after surgery, let alone for 3 to 6 months.
  - The recurrence rate of 10 to 12% is derived from just one small study and is likely to be inaccurate (due to definition issues). I would suggest the figure is derived from a suitable review article e.g. Kan *et al*., 2013 ^1^
  - Published recurrence data for collagenase are now available at 5 years (Peimer *et al.,* 2015 ^2^)
  - The complications for surgery and PNF and CCH are not comparable- the high rate of complications after CCH, although frequent, are transient. This should be emphasised.
- The Authors may consider mention that eventually, manipulation of genetic pathways rather than biochemical pathways will lead to the solution for this disease
- The tonometry is described as measuring “the hardness of the palmar tissue overlying the nodule” (page 8). Surely it measures the hardness of the nodule itself, not the palmar tissue overlying it.
- The inclusion criteria should include a single digit or palmar disease; otherwise the Michigan score will not distinguish disability from one or more cords
- Clarify the “range of motion” measurement. Would this be active or passive?

 

**POINTS THAT CANNOT BE IMPROVED**

- It remains to be shown, but is doubtful, that eradication of a nodule will prevent progression of disease in a different site- the nodule is probably not the seed from which the cord of DD grows, but just a localised manifestation of a diffuse tendency. By no means all nodules progress to symptomatic cords This point is crucial, yet seems glossed over in the introduction wherein the Authors make a rather enthusiastic pitch to imply that early treatment of nodules will eradicate the chance of the “risks” of other treatments being required. As a reviewer, I really feel uncomfortable with allowing this presumption to go unchallenged and insist on suitable justification or dilution.
- The clinical endpoint- treating nodules of DD before they progress, is laudable but unlikely to be cost effective. Perhaps 11% of people develop DD nodules. These nodules in their early stage might cause anxiety but rarely cause symptoms. It is unlikely that any health care system should view such treatment favourably when comparing the health gains with treating other more symptomatic or sinister conditions funding for which already challenges existing resources.
- The Authors aim in Study 2 to recruit patients with “up to 60 degrees”. This represents a substantial ***cord*** and does not represent a “ ***nodule***” nor “early DD”.  A cord of 60 degrees is likely to be symptomatic. Unless the proposed treatment removes the cord and thus corrects the deformity, entry into the trial of a patient with a combined deformity of 60 degrees will deprive the subject of treatment of a symptomatic condition. I respectfully advise that this inclusion extends beyond the purpose or capability of the study.
- The EQ5D questionnaire is worthless in DD as it has no sensitivity to change; it is the wrong PROM. The Authors have also employed the hand-specific Michigan scale  which is probably more suitable although I am not quite sure that references 37 and 38 robustly prove that it has been validated specifically for DD.

I have read this submission. I believe that I have an appropriate level of expertise to confirm that it is of an acceptable scientific standard, however I have significant reservations, as outlined above.

## References

[1] KanHJVerrijpFWHuisstedeBMHoviusSEvan NieuwenhovenCASellesRW: The consequences of different definitions for recurrence of Dupuytren's disease. *J Plast Reconstr Aesthet Surg*.2013;66(1) : 10.1016/j.bjps.2012.08.019 95-103 10.1016/j.bjps.2012.08.019 23137947

[2] PeimerCABlazarPColemanSKaplanFTSmithTLindauT: Dupuytren Contracture Recurrence Following Treatment With Collagenase Clostridium histolyticum (CORDLESS [Collagenase Option for Reduction of Dupuytren Long-Term Evaluation of Safety Study]): 5-Year Data. *J Hand Surg Am*.2015;40(8) : 10.1016/j.jhsa.2015.04.036 1597-605 10.1016/j.jhsa.2015.04.036 26096221

# Response

**Published:** 2017-11-15

**Authors:** Catherine Ball

**Affiliations:** Kennedy Institute University of Oxford, UK

We would like to thank Professor Isam Atroshi and Professor David Warwick for their supportive referee reports and helpful comments about our clinical trial protocol. In accordance with their suggestions, we have made some changes to the protocol text. We have clarified the degree of digital deformity in early Dupuytren’s disease for inclusion in the RIDD clinical trial. We also have now stated recurrence rates at 5 years. The technique for obtaining tonometry measurements is now described in more detail and we have explained how the photographs are used for consistent placement of the gauge. As requested, further details of the end-user survey concerning acceptance of injection therapy is now provided. We have also clarified that progression to surgery is a decision taken independently of the research team. Queries regarding bias and blinding have been addressed.

*STRONG POINTS*

*The Authors present a fascinating and plausible attempt to modify this enigmatic disease.*

*The study design is well-developed and appropriate for the matter in question.*

*POINTS TO IMPROVE*

*There does seem to be some misunderstanding of the difference between a nodule and a cord. Yet this distinction is essential. Surely the whole purpose of the study is to examine whether one can prevent a nodule from becoming a cord? This point needs to be fully addressed.*

 Response:

 We appreciate that nodules represent the early stage of disease, with subsequent development of cords. We will only be recruiting patients with distinct nodules and all injections will be administered intra-nodularly. Accurate assessment of development or extension of cords is very difficult and we will be measuring active and passive joint extension in the early disease stage RCT.

*In the Introduction and Discussion, there seems to me to have been just a little adornment in the quest to depict the need for this study: the portrayed risks of progression and complications of existing treatment, the alleged lack of cost effectiveness of treatment and the suggested need for prolonged hand therapy.*

*The facts in the introduction should therefore be qualified*

 Response:

 Please see above our amendment regarding potential complications of existing treatments. We will be assessing cost efficacy of adalimumab as part of our study.

*RCT evidence shows that splintage is not needed after surgery, let alone for 3 to 6 months.*

 Response:

 A recent Cochrane review ‘did not recommend routine use but that splinting should be considered on an individual basis’. (Rodrigues et al 2015). We agree that the needs of post-operative patients vary, with some patients requiring splintage but not routinely. This now reads: up to 6 months

*The recurrence rate of 10 to 12% is derived from just one small study and is likely to be inaccurate (due to definition issues). I would suggest the figure is derived from a suitable review article e.g. Kan et al., 2013 1*

 Response:

 Kan 2013 does not report recurrence rates of surgery alone. Recurrence rates from radiotherapy, fasciectomy, fasciotomy, collagenase injection and other treatments ranged from 2 to 80%. We quoted the data from a prospective randomised study (van Rijssen 2012), which compared 41 limited fasciectomy patients (125 joints) with 52 PNF patients (167 joints) after 5 years and found recurrence rates of 21% following limited fasciectomy and 85% following PNF.

 Page 3, column 1: We have amended the text to read: However, recurrence rates are high, at 5 years affecting 85% of patients treated with percutaneous needle aponeurotomy ^7^ and 47% of those treated with collagenase ^14^ . 

*Published recurrence data for collagenase are now available at 5 years (Peimer et al., 2015)*

 Response:

 Page 3, column 1: We have now cited comparable 5 year data and included the reference by Peimer et al (2015)

*The complications for surgery and PNF and CCH are not comparable- the high rate of complications after CCH, although frequent, are transient. This should be emphasised.*

 Response:

 Page 3, column 1: This has now been amended to read: over 70%, the majority being minor and mostly transient

*The Authors may consider mention that eventually, manipulation of genetic pathways rather than biochemical pathways will lead to the solution for this disease*

 Response:

 Gene therapy for non-life threatening conditions is some way from the clinic and we consider this to be outside the remit of our trial protocol.

*The tonometry is described as measuring “the hardness of the palmar tissue overlying the nodule” (page 8). Surely it measures the hardness of the nodule itself, not the palmar tissue overlying it.*

 Response:

 Page 8, column 2: We have amended this to read *“the hardness of the palmar tissue and the underlying nodule”*

*The inclusion criteria should include a single digit or palmar disease; otherwise the Michigan score will not distinguish disability from one or more cords*

 Response:

 By limiting recruitment to single digits the population would be very small and difficult to recruit.  We have included disease specific measures and functional measures.

*Clarify the “range of motion” measurement. Would this be active or passive?*

 Response:

 Page 8, column 2: This has now been amended to read: Active and passive range of movement.

 POINTS THAT CANNOT BE IMPROVED

*It remains to be shown, but is doubtful, that eradication of a nodule will prevent progression of disease in a different site- the nodule is probably not the seed from which the cord of DD grows, but just a localised manifestation of a diffuse tendency. By no means all nodules progress to symptomatic cords This point is crucial, yet seems glossed over in the introduction wherein the Authors make a rather enthusiastic pitch to imply that early treatment of nodules will eradicate the chance of the “risks” of other treatments being required. As a reviewer, I really feel uncomfortable with allowing this presumption to go unchallenged and insist on suitable justification or dilution.*

 Response:

 Whilst we agree that not all nodules progress to form cords with resultant flexion deformities of the digits, our published data show that there is an inverse correlation between the presence of nodules and disease severity (Verjee et al, JHS 2009. 34A: 1785-94).

*The clinical endpoint- treating nodules of DD before they progress, is laudable but unlikely to be cost effective. Perhaps 11% of people develop DD nodules. These nodules in their early stage might cause anxiety but rarely cause symptoms. It is unlikely that any health care system should view such treatment favourably when comparing the health gains with treating other more symptomatic or sinister conditions funding for which already challenges existing resources.*

 This study includes an economic evaluation to identify whether it is cost effective or not.

 Response:

 There is a clear plan to assess the cost efficacy of adalimumab. Please see tertiary objectives for the early disease RCT (Table 4)

*The Authors aim in Study 2 to recruit patients with “up to 60 degrees”. This represents a substantial cord and does not represent a “nodule” nor “early DD”.  A cord of 60 degrees is likely to be symptomatic. Unless the proposed treatment removes the cord and thus corrects the deformity, entry into the trial of a patient with a combined deformity of 60 degrees will deprive the subject of treatment of a symptomatic condition. I respectfully advise that this inclusion extends beyond the purpose or capability of the study.*

 Response:

 Whilst we agree that deformity of 60 degrees at a given joint would not be considered early stage disease, we are referring to up to 30 degrees at the proximal interphalangeal and metacarpophalangeal joints.

 Page 9, Table 6: We have clarified this section to read: Participants with early DD nodules shown or reported to progress in the previous 6 months, with a flexion deformity of ≤30° at the metacarpophalangeal and/or at the proximal interphalangeal joint, i.e. total flexion deformity of up to 60° for the digit being assessed.

 Individuals vary as to the level of deformity that they find acceptable and we will only recruit individuals who do not plan to undergo any elective procedure during the 18 month period of the trial. However, if the deformity progresses such that the participant and their treating clinicians decide to proceed with treatment, this will be recorded.

*The EQ5D questionnaire is worthless in DD as it has no sensitivity to change; it is the wrong PROM. The Authors have also employed the hand-specific Michigan scale which is probably more suitable although I am not quite sure that references 37 and 38 robustly prove that it has been validated specifically for DD.*

 Response:

 There are currently no data available for the EQ-5D-5L in assessing early DD. We are aware that whilst it may prove not to be sensitive, the EQ-5D-5L is a validated, generalised, health related quality of life questionnaire recommended by NICE as the accepted measure for conducting a cost-utility analysis. The EQ-5D instrument facilitates the generation of a utility score from a person’s health related quality of life.  A utility score refers to the preference that individuals have for any particular set of health outcomes.  The EQ-5D consists of five health state dimensions (mobility, self-care, usual activity, pain/discomfort, and anxiety/depression).  There are three levels of health status to choose from: no problems, some problems, major problems.  Each participant will value their present health at the date of questionnaire completion.   Participants will also complete a Visual Analogue Scale which will provide us with a value for the participant’s self-rated health at the time of survey completion.

 Page 11, column 2 has been amended accordingly.

 There are no published data relating to validation of the Michigan Hand Outcomes Questionnaire MHQ) in early DD. Reference 37 (Johnston et al 2008) showed that there was a strong correlation between in MHQ score and change in DD digital fixed flexion deformity. Reference 38 (Herweijer et al 2007) demonstrated that the MHQ can detect change in patients before and after surgery for DD. The MHQ has been shown to have high internal consistency which should be useful when exploring the natural course of DD, where changes occur slowly over time (Shauver and Chung 2013).

*Is the rationale for, and objectives of, the study clearly described?*

*Yes*

*Is the study design appropriate for the research question?*

*Yes*

*Are sufficient details of the methods provided to allow replication by others?*

*Yes*

*Are the datasets clearly presented in a useable and accessible format?*

*Yes*

*References*

*1. Kan HJ, Verrijp FW, Huisstede BM, Hovius SE, van Nieuwenhoven CA, Selles RW: The consequences of different definitions for recurrence of Dupuytren's disease.J Plast Reconstr Aesthet Surg. 2013; 66 (1): 95-103 PubMed Abstract |Publisher Full Text*

*2. Peimer CA, Blazar P, Coleman S, Kaplan FT, Smith T, Lindau T: Dupuytren Contracture Recurrence Following Treatment With Collagenase Clostridium histolyticum (CORDLESS [Collagenase Option for Reduction of Dupuytren Long-Term Evaluation of Safety Study]): 5-Year Data.J Hand Surg Am. 2015; 40 (8): 1597-605 PubMed Abstract | Publisher Full Text*

*Competing Interests: I have been a paid advisor on several occasions for SOBI, Pfizer and Actelion who market the drug Xiapex which is a treatment for Dupuytren’s Disease.*

*I have read this submission. I believe that I have an appropriate level of expertise to confirm that it is of an acceptable scientific standard, however I have significant reservations, as outlined above.*

 References to be added by authors

 Zhao, J.Z., et al., The Impact of Collagenase Clostridium histolyticum Introduction on Dupuytren Treatment Patterns in the United States. J Hand Surg Am, 2016. 41(10): p. 963-968.

 Thurston, A.J., Conservative surgery for Dupuytren's contracture. J Hand Surg Br, 1987. 12(3): p. 329-34.

 Gu, N.Y., et al., Eliciting health state utilities for Dupuytren's contracture using a discrete choice experiment. Acta Orthop, 2013. 84(6): p. 571-8.

# Peer review: Referee response for version 1

**DOI:** 10.21956/wellcomeopenres.12379.r23294

**Published:** 2017-06-26

**Authors:** Isam Atroshi

**Affiliations:** 1 Department of Clinical Sciences-Orthopedics, Lund University, Lund, Sweden

This is a well-written trial protocol. The trials address research questions that are important in the treatment of Dupuytren’s disease (DD).

 Introduction:

- The statement about the proportions of patients treated with surgery etc in the USA needs a reference and time frame because this changes over time.
- Postoperative hand therapy for 3-6 months sounds very long, usually not for the typical patient.
- To state that collagenase injection treatment has over 70% “complication rate” without specifying the type of complications is problematic. For people not familiar with collagenase it would sound that the treatment is not safe when in fact it is a very safe treatment because these “complications” are mostly local reactions such as swelling, minor hematomas, pain etc that resolve completely within days. Applying the same definition of complications to surgery would mean that all patients have complications.
- Second paragraph with rationale is very good.
- There are no details about how the “acceptability of injection therapy” survey was conducted.

Objectives and Methods:

- It is not entirely clear what is meant by “control the progression” of early DD.
- How do we know that nodule hardness is a measure of disease progression in patients with DD and joint contracture?
- Are there data about the reliability and validity of measuring “nodule hardness” and size?
- It would be helpful if a brief description of measuring nodule hardness is provided considering it is the primary outcome.
- Are there any data about responsiveness of the EQ-5D-5L in patients with DD?

Early DD RCT:                                  

- Definition of “Early” DD: 60 degrees of contracture in a finger is usually not considered early DD. It could be argued whether this is treatment or prevention of disease progression. Can the active injection result in partial or complete reduction of the contracture? What does the patient expect from the treatment?
- It is unclear whether the total flexion deformity of up to 60 degrees applies to 1 finger or multiple fingers, for example if a patient had contractures in the small, ring and middle fingers.
- Primary outcome: at what time point?
- How will the hand photos be evaluated and will it be done blinded?
- Is the injection painful (more painful than injecting saline)? Can this influence blinding?
- Does the injection cause local reaction like swelling, bruising etc, and if so how long do these changes persist? Can this influence blinding?
- It is stated that investigators will be able to discontinue participants on the basis of noncompliance such as not attending some visits; it would be important to avoid introducing risk of bias.
- How will the possible decision to proceed to surgery during the study be done?

I have read this submission. I believe that I have an appropriate level of expertise to confirm that it is of an acceptable scientific standard.

# Response

**Published:** 2017-11-15

**Authors:** Catherine Ball

**Affiliations:** Kennedy Institute University of Oxford, UK

We would like to thank Professor Isam Atroshi and Professor David Warwick for their supportive referee reports and helpful comments about our clinical trial protocol. In accordance with their suggestions, we have made some changes to the protocol text. We have clarified the degree of digital deformity in early Dupuytren’s disease for inclusion in the RIDD clinical trial. We also have now stated recurrence rates at 5 years. The technique for obtaining tonometry measurements is now described in more detail and we have explained how the photographs are used for consistent placement of the gauge. As requested, further details of the end-user survey concerning acceptance of injection therapy is now provided. We have also clarified that progression to surgery is a decision taken independently of the research team. Queries regarding bias and blinding have been addressed.

*This is a well-written trial protocol. The trials address research questions that are important in the treatment of Dupuytren’s disease (DD).*

*Introduction*

*The statement about the proportions of patients treated with surgery etc in the USA needs a reference and time frame because this changes over time.*

 Response:

 Page 3, Column 1: Reference now provided: Zhao et al. 2016

*Postoperative hand therapy for 3-6 months sounds very long, usually not for the typical patient.*

 Response:

 Page 3, Column 1: We agree that the duration of post-operative hand therapy varies, with some patients requiring more prolonged treatment. This now reads: up to 6 months

*To state that collagenase injection treatment has over 70% “complication rate” without specifying the type of complications is problematic. For people not familiar with collagenase it would sound that the treatment is not safe when in fact it is a very safe treatment because these “complications” are mostly local reactions such as swelling, minor hematomas, pain etc that resolve completely within days. Applying the same definition of complications to surgery would mean that all patients have complications.*

 Response:

 Page 3, Column 1: We have now amended this to read: over 70%, where the majority are relatively minor and transient, mainly comprising swelling, bruising and pain after collagenase injection.

*There are no details about how the “acceptability of injection therapy” survey was conducted.*

 Response:

 Page 3, Column 2: We now have included further details of the survey and the response rate. We have amended the text to read: We conducted an end user survey of 46 patients, 24 with early DD and 22 who had previously undergone surgery. Thirty-three patients (71.7%) responded 62.5% (n=15) and 82% (n=18) respectively from each group.

*Objectives and Methods:*

*It is not entirely clear what is meant by “control the progression” of early DD.*

 Response:

 Table 4, page 7: Progression of early disease is manifest as change in nodule hardness, nodule size or an increase in finger flexion deformity.  

*How do we know that nodule hardness is a measure of disease progression in patients with DD and joint contracture?*

*and*

*Are there data about the reliability and validity of measuring “nodule hardness” and size?*

 Response:

 We agree that nodule hardness has not been shown to correlate with joint contracture. Relying on progression of finger flexion as a measure of disease progression would have resulted in an unacceptably long follow up period of 3-5 years. We selected nodule hardness as the primary outcome measure in line with the study by Ketchum et al 2000, who assessed the efficacy of local steroid injection into the nodule. However, whilst Ketchum et al relied on subjective assessment of hardness, we will use tonometry to obtain a quantitative measure. We found that tonometry readings were lower in healthy volunteers compared to people with DD – please refer to the section on Sample size calculation, page 7. We also referenced a publication by Thurston (1987) who used tonometry and reported softening of the palm following excision of Dupuytren’s tissue. We will also use ultrasound scan to assess the size of Dupuytren’s nodules but as there are no previous data using this modality for Dupuytren’s disease we have chosen this parameter as a secondary objective. Measurement of surface area of nodules has been shown not to correlate with disease progression Lanting (2016).

Ketchum LD, Donahue TK: The injection of nodules of Dupuytren's disease with triamcinolone acetonide. *J Hand Surg Am.* 2000;25(6):1157–62. 11119679 10.1053/jhsu.2000.18493

*It would be helpful if a brief description of measuring nodule hardness is provided considering it is the primary outcome.*

 Response:

 Page 11, Column 1: This section has been amended to read: Tissue hardness will be measured with a tonometer, ensuring that the gauge is balanced perpendicular to the skin without application of external pressure by the observer. Five readings will be obtained on each occasion. A photograph obtained at base line marking the spot to be measured will be used for all subsequent measurement to ensure consistency of placement of the tonometer. Tonometry has been used to evaluate skin compliance before and after surgery (Thurston 1987) and reported softening of the palm following excision of Dupuytren’s tissue.

*Are there any data about responsiveness of the EQ-5D-5L in patients with DD?*

 Response:

 Page 11, Column 2: The EQ-5D-5L has been used to calculate health state utilities (Gu 2013) in individuals with more advanced DD. This has now been added the section Health Utilities in Clinical outcome measures.

*Early DD RCT:*

*Definition of “Early” DD: 60 degrees of contracture in a finger is usually not considered early DD. It could be argued whether this is treatment or prevention of disease progression. Can the active injection result in partial or complete reduction of the contracture? What does the patient expect from the treatment?*

 Response:

 Page 9, Table 6: Whilst we agree that deformity of 60 degrees at a given joint would not be considered early stage disease, we are referring to up to 30 degrees at the proximal interphalangeal and metacarpophalangeal joints.

 We have clarified this section to read: Participants with early DD nodules shown or reported to progress in the previous 6 months, with a flexion deformity of ≤30° at the metacarpophalangeal and/or at the proximal interphalangeal joint, i.e. total flexion deformity of up to 60° for the digit being assessed.

*It is unclear whether the total flexion deformity of up to 60 degrees applies to 1 finger or multiple fingers, for example if a patient had contractures in the small, ring and middle fingers.*

 Response:

 Page 9, Table 6: This refers to a single digital flexion deformity of the digit that we are assessing as clarified above.

*Primary outcome: at what time point?*

 Response:

 Page 8: The primary outcome is measured over time as described in Table 5, with the final measurement being taken at 18 months.

*How will the hand photos be evaluated and will it be done blinded?*

 Response:

 In the Tissue response RCT the photographs will be used to evaluate healing and quality of scarring.

 Page 11, column 1: In the early DD RCT they will not be evaluated but used to ensure consistent placement of the tonometer.

*Is the injection painful (more painful than injecting saline)? Can this influence blinding?*

 Response:

 It is not known whether injection of adalimumab into a nodule is more painful than saline although when administered subcutaneously there were no significant differences in the pain scores (Furst  2003). Pain will not influence blinding as each participant will receive the same treatment (adalimumab or placebo) throughout the course of the study.

  Furst, D.E., et al., Adalimumab, a fully human anti tumor necrosis factor-alpha monoclonal antibody, and concomitant standard antirheumatic therapy for the treatment of rheumatoid arthritis: results of STAR (Safety Trial of Adalimumab in Rheumatoid Arthritis). The Journal of Rheumatology, 2003. **30**(12): p. 2563-2571.

*Does the injection cause local reaction like swelling, bruising etc, and if so how long do these changes persist? Can this influence blinding?*

 Response:

 The participant will be blinded to the treatment received and, as explained above, will receive the same treatment (adalimumab or placebo) throughout. Therefore, the duration of any local adverse effects will not influence participant blinding. All assessments will be performed by a blinded observer before administration of the injection.

*It is stated that investigators will be able to discontinue participants on the basis of noncompliance such as not attending some visits; it would be important to avoid introducing risk of bias.*

 Response:

 Injections will be discontinued if the participant withdraws from the trial or if the investigator considers it necessary for any reason, including pregnancy, adverse events, significant protocol deviation or non-compliance with study procedures. Compliance will be defined as attending not less than 75% of injection visits, i.e. 3 visits. We will endeavour to continue to follow up the participants with their consent following withdrawal.

*How will the possible decision to proceed to surgery during the study be done?*

 Response:

 The participant will follow the normal clinical pathway. This now reads: Progression to treatment will be determined by clinicians who are not part of the trial team. 

*Is the rationale for, and objectives of, the study clearly described?*

*Yes*

*Is the study design appropriate for the research question?*

*Yes*

*Are sufficient details of the methods provided to allow replication by others?*

*Yes*

*Are the datasets clearly presented in a useable and accessible format?*

*Yes*

*Competing Interests: No competing interests were disclosed.*

*I have read this submission. I believe that I have an appropriate level of expertise to confirm that it is of an acceptable scientific standard.*

 References to be added by authors

 Zhao, J.Z., et al., The Impact of Collagenase Clostridium histolyticum Introduction on Dupuytren Treatment Patterns in the United States. J Hand Surg Am, 2016. 41(10): p. 963-968.

 Thurston, A.J., Conservative surgery for Dupuytren's contracture. J Hand Surg Br, 1987. 12(3): p. 329-34.

 Gu, N.Y., et al., Eliciting health state utilities for Dupuytren's contracture using a discrete choice experiment. Acta Orthop, 2013. 84(6): p. 571-8.
